# Effect of subclinical esketamine on NLRP3 and cognitive dysfunction in elderly ischemic stroke patients

**DOI:** 10.1515/med-2025-1193

**Published:** 2025-05-22

**Authors:** Lang Wang, Baoxin Ma, Jianping Zhang

**Affiliations:** Department of Anesthesiology Operation Room, Zhongshan Hospital Xiamen University, School of Medicine, Xiamen University, Fujian, 361004, P. R. China; Department of Anesthesiology Operation Room, Zhongshan Hospital Xiamen University, School of Medicine, Xiamen University, 201-209 Hubin South Road, Siming District, Fujian, 361004, P. R. China

**Keywords:** esketamine, ischemic stroke, NLRP3, MMSE, cognitive dysfunction

## Abstract

**Objective:**

This study investigates the effects of subclinical doses of esketamine on serum NLRP3 levels and early cognitive dysfunction in elderly ischemic stroke patients after neurointerventional procedures under general anesthesia.

**Methods:**

A prospective cohort study included 120 elderly ischemic stroke patients undergoing general anesthesia from January 2022 to September 2023. The esketamine group received 0.25 mg/kg of esketamine. Serum levels of NLRP3, C-reactive protein, interleukin-6 (IL-6), IL-1β, and IL-17 were measured before surgery and 24 h postoperatively. Cognitive dysfunction was assessed using the Mini-Mental State Examination (MMSE).

**Results:**

At 24 h postoperatively, the esketamine group had significantly higher MMSE scores (*p* < 0.05) and lower serum levels of NLRP3, IL-17, and IL-6. Pearson’s correlation showed a link between NLRP3 levels and cognitive outcomes. Logistic regression identified heart rate, mean arterial pressure, preoperative NLRP3, IL-6, IL-17, and esketamine treatment as risk factors for cognitive dysfunction.

**Conclusion:**

Subclinical doses of esketamine might reduce postoperative cognitive dysfunction risk and offer neuroprotection, presenting potential therapeutic options for elderly ischemic stroke patients.

## 1 Introduction

Stroke remains a leading cause of long-term disability, affecting approximately 13.7 million individuals globally and contributing to 5.5 million deaths annually [1,2,3,4]. Clinically, stroke is categorized as either ischemic or hemorrhagic, with ischemic stroke accounting for nearly 85% of all cases [5]. In recent years, neurointerventional therapies performed under general anesthesia – particularly mechanical thrombectomy – have emerged as cornerstone treatments for ischemic stroke. Mechanical thrombectomy, in particular, is considered a key method for treating ischemic stroke [6]. Despite their benefits, these procedures are frequently associated with postoperative cognitive dysfunction (POCD), which significantly impairs patients’ quality of life and functional recovery, which can have a substantial impact on the quality of life and functional outcomes of the patients [7].

Esketamine, a stereoisomer of ketamine, is a dissociative anesthetic agent that is widely used for analgesia and anesthesia [8]. Emerging evidence suggests that esketamine may possess neuroprotective properties, partly by modulating inflammation-related pathways such as STING/TBK1 and HDAC3/NF-κB/COX1 [9]. There are also reports showing that esketamine improves post-stroke anxiety by modulating the ischemic cortical microglial HDAC3/NF-κB/COX1 inflammatory signaling pathway [10]. Moreover, esketamine appears to regulate microglial activation and inflammatory responses, thus contributing to reduced neuroinflammation. In addition to neuroprotective effects, esketamine has been shown to exert anti-inflammatory, which may be mediated through its modulation of microglial activity and inflammatory signaling pathways [11]. Han et al. found the anti-inflammatory effects of esketamine in orthopedic surgery and protection against postoperative cognitive function and pain control [12].

NLRP3 is a recently discovered key mediator of neuroinflammation closely associated with inflammatory response [13]. Increasing evidence suggested that NLRP3 played a significant physiological and pathological role in the development of ischemic stroke [14]. Activation of autophagy can inhibit the activation of NLRP3 inflammasomes and alleviate sevoflurane-induced cognitive dysfunction in elderly rats [15]. This indicates that NLRP3 plays an important role in post-anesthesia cognitive function. However, currently, there is a relative lack of research on the expression of NLRP3 in patients and clinical studies.

Therefore, the purpose of this study was to investigate the effects of subclinical doses of esketamine on serum NLRP3 levels in elderly patients with ischemic stroke after general anesthesia neurointerventional procedures. Additionally, the study aimed to explore the correlation between NLRP3 levels and early cognitive dysfunction in this patient population and to explore risk factors for POCD in patients. Understanding the impact of esketamine on NLRP3 and its association with cognitive dysfunction could provide valuable insights into potential therapeutic strategies to prevent or mitigate POCD in elderly stroke patients.

## 2 Methods

### 2.1 Study population

This prospective observational cohort study included 120 elderly patients with ischemic stroke who underwent general anesthesia for neurointerventional procedures at our hospital from January 2022 to September 2023. The inclusion criteria were as follows: (1) diagnosis with ischemic stroke based on the “2018 Chinese Guidelines for the Diagnosis and Treatment of Acute Ischemic Stroke” [16] using computed tomography or magnetic resonance imaging; (2) aged ≥60 years, first occurrence of stroke, and admission within 12 h of symptom onset; (3) American Society of Anesthesiologists physical status classification ≤3. The exclusion criteria were as follows: (1) patients who did not require surgical treatment; (2) patients with hemorrhagic stroke; (3) patients unable to undergo scale assessments within 24 h after surgery; (4) patients with severe infection, severe liver or kidney dysfunction, or malignant tumors; (5) patients with epilepsy, Parkinson’s disease, or other neurological or psychiatric disorders; (6) patients who received anticoagulant therapy within 3 months before the study; and (7) patients with an education level of <6 years. All patients maintained spontaneous breathing during the general anesthesia. The anesthesia protocol for the control group included the use of dexmedetomidine (20 µg) and glycopyrrolate (0.2 mg) for induction, maintenance of spontaneous breathing, oxygen flow rate of 2 L/min, placement of a nasopharyngeal airway, monitoring of end-tidal carbon dioxide partial pressure, and maintenance anesthesia with propofol (0.5–1 mg/kg). In the esketamine group, patients received an additional subclinical dose of esketamine (0.25 mg/kg) in addition to the standard anesthesia protocol. Furthermore, all patients received treatments including antiplatelet aggregation, circulation improvement, neuroprotection, free radical scavenging, and statin therapy according to the “2018 Chinese Guidelines for the Diagnosis and Treatment of Acute Ischemic Stroke.” Written informed consent was obtained from all patients. This study was approved by our hospital’s ethics committee.

### 2.2 Enzyme-linked immunosorbent assay

The levels of NLRP3, C-reactive protein (CRP), interleukin-6 (IL-6), IL-1β, and IL-17 in serum were measured using ELISA. In brief, fasting venous blood samples (5 mL) were collected from all patients. The collected blood samples were centrifuged at 2,000*g* for 15 min. After centrifugation, commercially available ELISA kits (NLRP3 MBS9137465 MyBioSource, CRP MBS177184 MyBioSource, IL-6 MBS175877 MyBioSource, IL-1β MBS2021180 MyBioSource, IL-17 MBS2019491 MyBioSource) were used to measure the levels of NLRP3, CRP, IL-6, IL-1β, and IL-17 in strict accordance with the manufacturer’s instructions. The levels of these serum biomarkers were measured before surgery and 24 h after surgery.

### 2.3 Outcome measures

Demographic baseline parameters of the two groups, including age, sex, body mass index (BMI), systolic blood pressure (SBP), diastolic blood pressure (DBP), fasting plasma glucose, and comorbidities (hypertension, diabetes, coronary heart disease), were collected. In addition, surgical duration, anesthesia duration, intraoperative blood loss, heart rate (HR), mean arterial pressure (MAP), and SPO2 at induction (T1), intubation (T2), and extubation (T3) were recorded during the surgery for both groups. Furthermore, cognitive dysfunction was evaluated 24 h after surgery using the Mini-Mental State Examination (MMSE) scale. MMSE scores <27 were defined as the presence of POCD [17].

### 2.4 Statistical analysis

Data analysis was performed using SPSS 25.0 software (IBM, Armonk, NY, USA). The normality of the data was confirmed using the Kolmogorov–Smirnov test. Normally distributed data are presented as mean ± standard deviation, while non-normally distributed data are presented as median (range). The Mann–Whitney test or Student’s *t*-test was used for comparisons between the two groups. The chi-square test was used for proportions. Pearson’s and Sparman’s correlation analysis was performed to assess the correlations between serum inflammatory factors and between NLRP3 and clinical characteristics in patients. Receiver operating characteristic (ROC) curve analysis was conducted to evaluate the diagnostic value of serum NLRP3 in predicting POCD. Furthermore, logistic regression analysis was performed to identify the risk factors for POCD in elderly patients with ischemic stroke after general anesthesia. A *p*-value < 0.05 was considered statistically significant.

## 3 Results

### 3.1 Baseline characteristics of all patients

In this prospective observational cohort study, during the study period, a total of 148 patients were initially screened for eligibility. Of these, 120 patients met the inclusion criteria and were enrolled in the study, while 28 patients were excluded based on the following exclusion criteria: patients who did not require surgical treatment (*n* = 7); patients with hemorrhagic stroke (*n* = 3); patients unable to undergo scale assessments within 24 h after surgery (*n* = 8); patients with severe infection, severe liver or kidney dysfunction, or malignant tumors (*n* = 3); patients with epilepsy, Parkinson’s disease, or other neurological or psychiatric disorders (*n* = 4); patients who received anticoagulant therapy within 3 months prior to the study (*n* = 1); and patients with an education level of <6 years (*n* = 2).

We included two groups of 120 patients, with 63 patients in the control group and 57 patients in the esketamine group. No significant adverse effects were observed in the esketamine group during or after the procedure. After comparing the demographic data, we found no significant differences between the two groups in terms of age, sex, BMI, and comorbidities. In terms of intraoperative clinical data, the HR and MAP of patients in the esketamine group were significantly lower at T3 compared to the control group (*p* < 0.05). No differences were observed between the two groups in terms of surgical duration, anesthesia duration, HR, MAP, and SPO2 at other time points. Furthermore, we found that the MMSE score at 24 h postoperatively was significantly higher in the esketamine group compared to the control group (Table 1, *p* < 0.05).

**Table 1:** Basic characteristics of all patients

Variable	Esketamine group, *n* = 63	Control group, *n* = 57	*p*
Age, years	67 (60–77)	66 (67–77)	0.790
Sex, female (%)	27 (42.9)	30 (52.6)	0.170
BMI	22.15 ± 2.11	22.26 ± 2.36	0.788
SBP (mmHg)	122.25 ± 15.93	126.42 ± 18.40	0.186
DBP (mmHg)	90 (62–107)	90 (63–106)	0.737
Hypertension, *n* (%)	41 (65.1)	35 (61.4)	0.587
Diabetes, *n* (%)	15 (23.8)	11 (19.3)	0.439
Coronary heart disease, *n* (%)	6 (9.5)	4 (7.0)	0.521
Intraoperative blood loss (mL)	62.27 ± 12.62	61.48 ± 11.12	0.716
Anesthesia time (min)	120 (85–142)	119 (86–143)	0.642
Operative time (min)	84.79 ± 9.76	87.54 ± 10.14	0.132
MMSE 24 h after surgery	28 (17–30)	27 (17–30)	0.026
**T1**			
HR/min	84.96 ± 4.38	85.31 ± 4.30	0.661
MAP (mm Hg)	93.47 ± 7.76	92.64 ± 7.16	0.544
SPO2 (%)	97.81 ± 0.60	97.81 ± 0.53	0.961
**T2**			
HR/min	67.28 ± 3.87	67.81 ± 4.40	0.479
MAP (mm Hg)	80.33 ± 5.86	78.31 ± 6.57	0.077
SPO2 (%)	98.27 ± 0.55	98.32 ± 0.57	0.663
**T3**			
HR/min	74.85 ± 3.98	77.97 ± 5.38	0.032
MAP (mm Hg)	82.84 ± 6.18	87.11 ± 5.91	<0.001
SPO2 (%)	97.98 ± 0.61	97.95 ± 0.56	0.762

### 3.2 Differential expression of serum biomarkers in elderly patients with ischemic stroke after surgery

**Figure 1:**
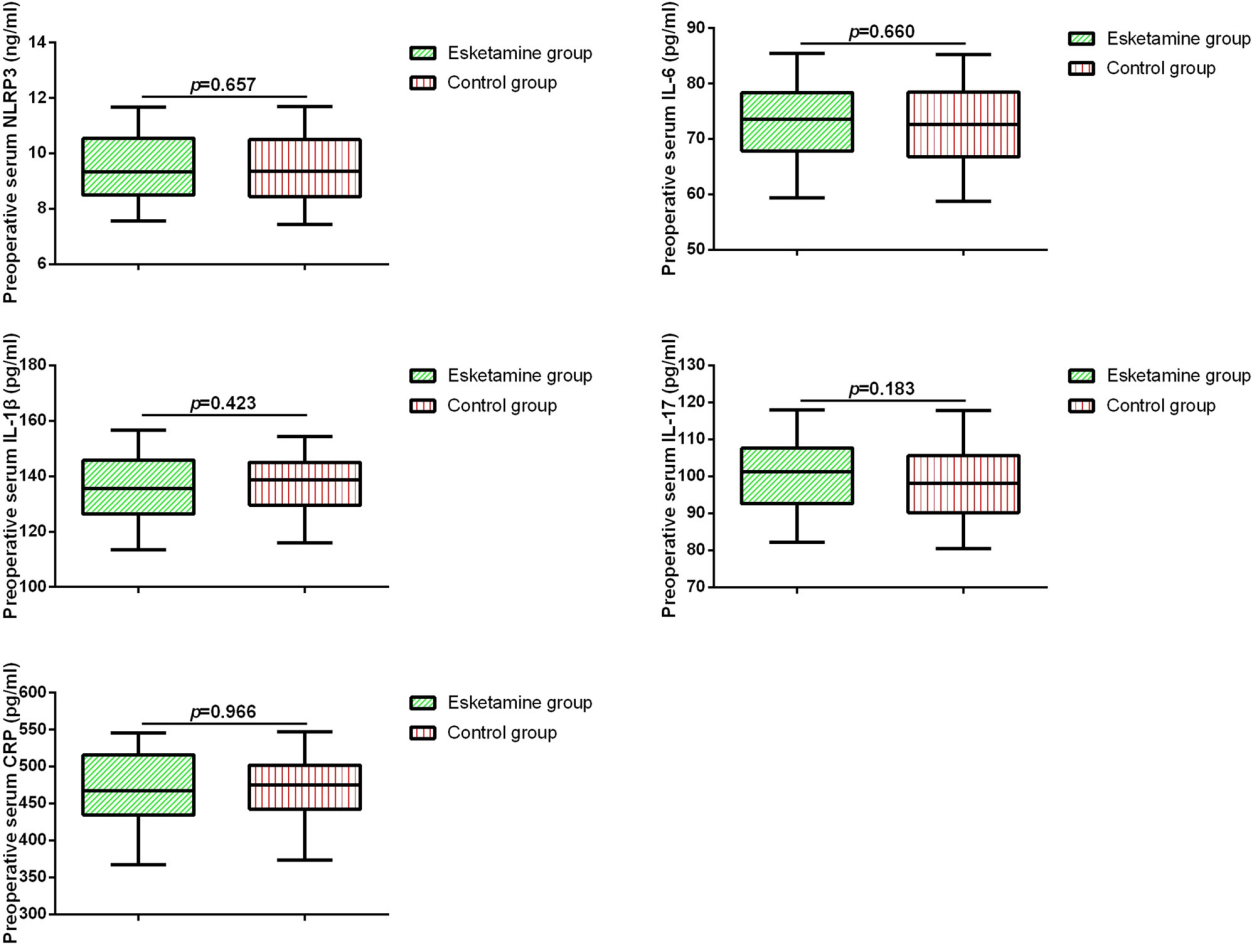
Preoperative serum biomarkers in elderly patients with ischemic stroke.

**Figure 2:**
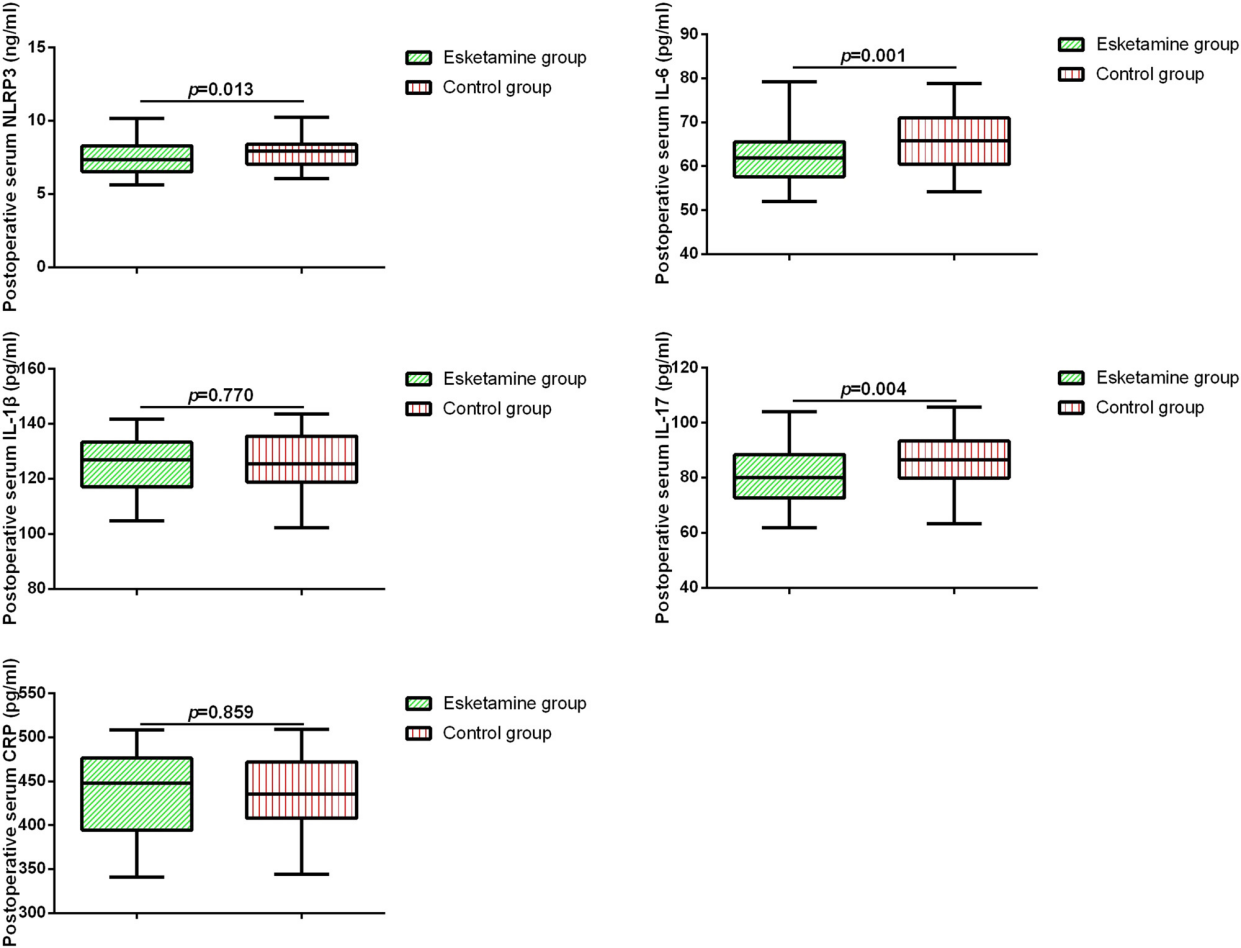
Serum biomarkers in elderly patients with ischemic stroke 24 h after surgery.

Next, we compared the expression levels of serum NLRP3, IL-6, IL-1β, IL-17, and CRP in the two groups of patients before surgery and 24 h postoperatively. As shown in Figures 1 and 2, there were no significant differences in the levels of serum cytokines between the two groups before surgery. However, after 24 h of surgery, patients in the esketamine group exhibited significantly lower levels of serum NLRP3, IL-17, and IL-6 compared to the control group. Pearson’s correlation analysis revealed a positive correlation between serum NLRP3 levels and IL-17 levels as well as IL-6 levels (Table 2).

**Table 2:** Correlation analysis among cytokines at 24 h postoperatively

	CRP	IL-6	IL-1β	IL-17	NLRP3
**CRP**					
Pearson’s correlation	1	−0.052	−0.092	0.003	−0.006
*p*		0.576	0.305	0.970	0.949
**IL-6**					
Pearson’s correlation	−0.052	1	−0.033	0.070	0.273
*p*	0.576		0.722	0.445	0.003
**IL-1β**					
Pearson’s correlation	−0.092	−0.033	1	0.102	0.023
*p*	0.305	0.722		0.268	0.804
**IL-17**					
Pearson’s correlation	0.003	0.070	0.102	1	0.145
*p*	0.970	0.445	0.268		0.115
**NLRP3**					
Pearson’s correlation	−0.006	0.273	0.023	0.145	1
*p*	0.949	0.003	0.804	0.115	

### 3.3 Correlation between NLRP3 levels and clinical characteristics of patients

We further analyzed the relationship between serum NLRP3 levels at 24 h postoperatively and the clinical characteristics of elderly patients with ischemic stroke. Spearman’s analysis showed a positive correlation between serum NLRP3 levels and MAP at the T3 time point, with Spearman’s coefficients of 0.309 (*p* < 0.05). In addition, we observed a negative correlation between serum NLRP3 levels and MMSE scores, with Spearman’s coefficients of −0.432 (Table 3, *p* < 0.05). This indicates that there is an association between serum NLRP3 levels and the clinical characteristics and postoperative cognitive status of patients.

**Table 3:** Correlation between serum NLRP3 levels and the clinical data of all patients

Variable	NLRP3	
	Spearman’s correlation	*p*
Intraoperative blood loss	−0.034	0.709
Anesthesia time	−0.055	0.547
Operative time	−0.011	0.906
T1HR	0.072	0.437
T2HR	−0.066	0.476
T3HR	−0.055	0.553
T1MAP	−0.040	0.668
T2MAP	−0.066	0.474
T3MAP	0.309	0.001
T1SPO2	−0.026	0.774
T2SPO2	0.035	0.708
T3SPO2	−0.107	0.231
MMSE	−0.432	<0.001

### 3.4 Diagnostic value of NLRP3 for POCD in elderly patients with ischemic stroke

**Figure 3:**
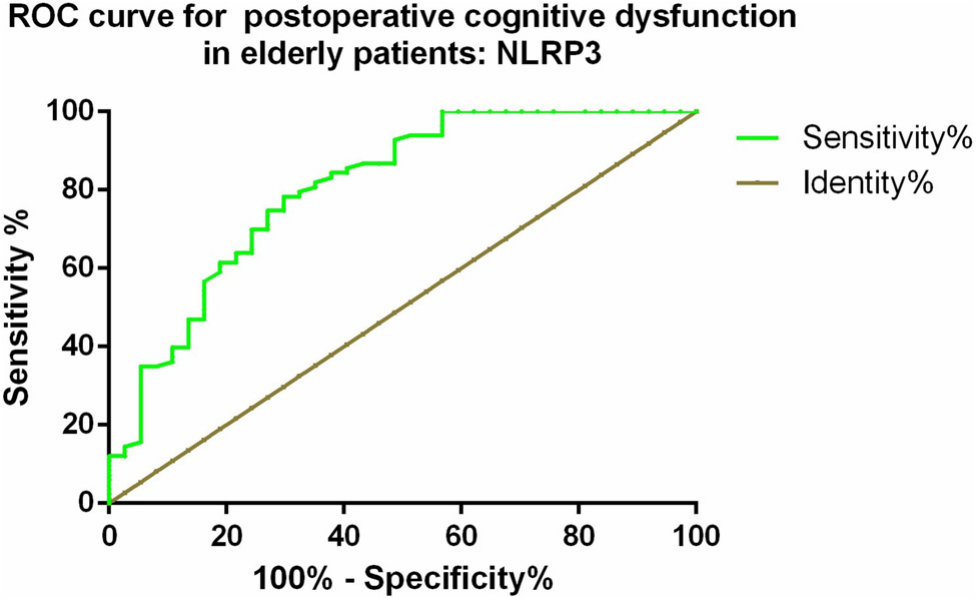
ROC curve of NLRP3 for POCD in elderly patients with ischemic stroke after surgery.

Based on the postoperative 24 h MMSE scores, we divided elderly patients with ischemic stroke into a cognitive dysfunction group (MMSE < 27, *n* = 37) and a non-cognitive dysfunction group (MMSE > 27, *n* = 83). ROC curve analysis was performed to determine the diagnostic value of serum NLRP3 levels for POCD in patients. The results showed that serum NLRP3 levels had a certain diagnostic value for POCD in elderly patients with ischemic stroke, with an AUC of 0.805, a cutoff value of 7.92 ng/mL, sensitivity of 73.0%, and specificity of 74.7% (Figure 3).

### 3.5 Logistic regression analysis of risk factors for POCD in elderly patients with ischemic stroke

Finally, we conducted a logistic regression analysis to identify the risk factors for POCD in all elderly patients with ischemic stroke. The results revealed that HR at T3, MAP at T3, preoperative NLRP3, IL-6, IL-17 levels, and esketamine treatment were all risk factors for cognitive dysfunction in elderly patients undergoing general anesthesia for ischemic stroke (Table 4).

**Table 4:** Logistic regression of risk factors for POCD

Variables	Wald	Odds ratio	95% CI	*p*
Operative time	2.278	0.953	0.895–1.015	0.131
Anesthesia time	1.175	1.021	0.984–1.059	0.278
Intraoperative blood loss	0.002	1.001	0.952–1.053	0.963
T1HR	1.1.03	1.082	0.928–1.263	0.314
T2HR	0.545	0.941	0.800–1.106	0.460
T3HR	12.968	1.354	1.148–1.597	<0.001
T1MAP	0.573	0.967	0.885–1.055	0.449
T2MAP	0.001	1.001	0.902–1.111	0.984
T3MAP	20.906	1.430	1.227–1.667	<0.001
T1SPO2	0.004	0.963	0.310–2.990	0.948
T2SPO2	1.035	0.560	0.183–1.712	0.309
T3SPO2	3.106	2.554	0.900–7.245	0.078
Preoperative NLRP3	16.901	0.249	0.128–0.483	<0.001
Preoperative IL-6	14.288	0.792	0.702–0.894	<0.001
Preoperative IL-1β	1.608	0.971	0.919–1.027	0.302
Preoperative IL-17	9.827	0.907	0.853–0.964	0.002
Preoperative CRP	0.143	1.003	0.990–1.016	0.705
Esketamine treatment	8.268	0.301	0.133–0.682	0.004

## 4 Discussion

POCD is a common complication among elderly patients undergoing surgery, often presenting as impairments in memory, attention, and executive function. Additionally, this pathological condition not only leads to a decrease in quality of life but also signifies disease progression [18]. Early detection and intervention are therefore critical for improving neurological and functional outcomes in this population. In this study, we found a significant increase in serum NLRP3 levels at 24 h postoperatively in elderly patients with ischemic stroke who developed POCD. Furthermore, NLRP3 and esketamine were identified as risk factors for cognitive dysfunction in elderly patients undergoing general anesthesia.

Previous studies have extensively reported the use of esketamine alone or in combination with other agents during anesthesia induction, demonstrating improved patient compliance and effective sedation outcomes [19,20]. In recent years, subclinical doses of esketamine have attracted increasing attention due to their potential neuroprotective properties. These doses are defined as being lower than those used for general anesthesia but still capable of exerting therapeutic effects. For example, Li et al. administered 0.2 mg/kg of esketamine during anesthesia induction in elderly patients undergoing unilateral total knee arthroplasty under general anesthesia. The results showed that subclinical doses of esketamine better maintained hemodynamic stability and had no adverse effects on early postoperative recovery quality [21]. Similarly, Zhang et al. found that subanesthetic doses of esketamine relieved pain in the postanesthesia care unit after laparoscopic cholecystectomy, although it may delay aesthetic recovery [22]. Consistent with these findings, no significant adverse events were observed in our esketamine group. This suggested that subclinical doses of esketamine could be safely administered to elderly ischemic stroke patients undergoing neurointerventional procedures. Additionally, Ma et al. used 0.25 mg/kg of esketamine during anesthesia induction in elderly patients undergoing general anesthesia for gastrointestinal tumor surgery, which is consistent with the dose used in our study. In their study, they found that low-dose esketamine reduced the occurrence of delayed neurocognitive recovery in elderly patients undergoing gastrointestinal tumor surgery under general anesthesia [23]. Our study supported this conclusion, suggesting that the use of subclinical doses of esketamine in elderly patients with ischemic stroke might provide neuroprotection and potentially mitigate POCD.

Inflammation following ischemic stroke is considered to be an unavoidable pathological process in post-ischemic brain injury [24,25]. The dynamic release of various pro- and anti-inflammatory cytokines in the brain after stroke may influence the progression of cerebral infarction and contribute to specific cognitive symptoms, including memory impairment, attention deficits, and executive dysfunction. These inflammatory factors are therefore regarded as important biomarkers of stroke pathogenesis and prognosis [26]. Morrison et al. demonstrated that elevated IL-17A levels were associated with poorer executive function and attention in middle-aged individuals, suggesting a direct link between IL-17A and specific cognitive domains [27]. Similarly, Baier et al. found that IL-6 knockout mice exhibited impaired memory in both hippocampus-dependent and independent tasks, indicating that IL-6 plays a critical role in memory processes [28]. Furthermore, Danielski et al. showed that inhibiting the NLRP3 inflammasome reduced neuroinflammation, oxidative stress, and cognitive impairment in a sepsis model, highlighting the importance of NLRP3 in mediating inflammation-related cognitive dysfunction [29]. These studies collectively suggested that specific inflammatory factors were closely associated with distinct cognitive symptoms, underscoring the potential of targeting these pathways to mitigate POCD.

Recent research has indicated that neuroinflammation may be a common precursor to cognitive decline, and inflammatory-related factors, microglial activation, and the interaction between the peripheral immune system and the central nervous system are all involved in cognitive impairment. Therefore, limiting acute neuroinflammation might improve cognitive function and significantly enhance patient prognosis [30]. The NLRP3 inflammasome plays a central role in regulating inflammatory response. Activation of NLRP3-related signaling pathways has been implicated in various neurological disorders, including cognitive dysfunction [31,32]. Elevated NLRP3 levels have been associated with increased inflammation and neuronal damage [33]. Inhibiting NLRP3 signaling has been shown to improve POCD and neuroinflammatory responses in elderly mice [34]. Hence, studying the relationship between NLRP3 and early cognitive dysfunction in elderly patients with ischemic stroke after neurointerventional procedures is of great importance. In this study, we found that serum NLRP3 levels were associated with clinical characteristics and postoperative cognitive outcomes in patients. Furthermore, NLRP3 was identified as an independent risk factor for cognitive dysfunction in elderly patients undergoing general anesthesia. While our findings support the short-term benefits of esketamine, further studies are needed to determine whether its effects on NLRP3 levels and cognitive function are sustained over time. These insights may help establish esketamine as a long-term therapeutic strategy and contribute to the development of targeted interventions for POCD. NLRP3 is not only a key regulator of inflammation but also a critical mediator of pyroptosis, a form of programmed cell death characterized by the activation of caspase-1, cleavage of gasdermin D (GSDMD), and release of pro-inflammatory cytokines such as IL-1β and IL-18. Pyroptosis has been increasingly recognized as a significant contributor to neuroinflammation and cognitive decline, particularly in the context of POCD [35,36]. Recent studies have explored the potential of esketamine, the S-enantiomer of ketamine, in modulating pyroptosis and its associated pathways. Li et al. found that esketamine exerted neuroprotective effects in aged rats with POCD by inhibiting the STING/TBK1 pathway and downregulating pyroptosis-associated proteins such as cleaved caspase-1 and IL-18 [9]. Similarly, Zhou et al. showed that low-dose esketamine downregulated key pyroptosis markers, including NLRP3, cleaved caspase-1, IL-1β, and GSDMD-N, in hippocampal tissues and microglial cells [37]. Despite these promising findings, evidence regarding the role of esketamine in regulating pyroptosis in ischemic stroke and POCD remains limited. Therefore, further studies are needed to explore these mechanisms in elderly patients undergoing neurointerventional procedures, with the aim of identifying new therapeutic targets to mitigate cognitive decline and improve patient outcomes.

Certain limitations to our study should be acknowledged. First, the sample size was relatively small. Second, our analysis only assessed a limited number of serum biomarkers. Additionally, our study did not explore the specific mechanisms by which esketamine might influence NLRP3 levels, such as through NMDA receptor antagonism or modulation of microglial activity. Future mechanistic studies are needed to elucidate these pathways. Finally, while no significant adverse effects were observed in the esketamine group, our study was not specifically designed to evaluate rare or long-term adverse effects of esketamine. Future studies with larger sample sizes and longer follow-up periods are needed to further assess the safety profile of esketamine in this patient population.

## 5 Conclusion

In conclusion, our study found that the use of subclinical doses of esketamine might provide neuroprotection and potentially reduced the risk of POCD. Furthermore, NLRP3 and the use of esketamine were identified as relevant factors for the development of cognitive dysfunction in elderly patients with ischemic stroke undergoing neurointerventional surgery. This study might provide new therapeutic targets and comprehensive treatment strategies for POCD.
